# Supplementary material for: Forest Type and Climate Outweigh Soil Bank in Shaping Dynamic Changes in Macrofungal Diversity in the Ancient Tree Park of Northeast China
Source: J Fungi (Basel). 2023 Aug 17;9(8):856. doi: 10.3390/jof9080856 (PMC10455530; doi:10.3390/jof9080856)
Supplement: Supplementary file 1 [file jof-09-00856-s001.zip › jof-2500271-supplementary.pdf]

Table S1. Macrofungi were collected in three forest types of Changbai Mountain Ancient Tree Park

Species	Family	Genus	B	C	M	N	ML	SN	GenBank Number
Agaricus alboumbonatus	Agaricaceae	*Agaricus*	√		√	2	SS	15896-15905	OR364536
Agaricus campestris	Agaricaceae	*Agaricus*	√			2	SS	17259-17262	
Agaricus daliensis	Agaricaceae	*Agaricus*			√	1	SS	16598-16601	OR364537
Agaricus indistinctus	Agaricaceae	*Agaricus*	√		√	2	SS	10546-10571	
Agaricus sylvaticus	Agaricaceae	*Agaricus*			√	3	SS	HMJAU63963	OR364538
Agrocybe erebia	Strophariaceae	*Agrocybe*			√	1	SS	27633-27638	
Agrocybe firma	Strophariaceae	*Agrocybe*	√		√	4	SS	HMJAU63986	OR364539
Agrocybe praecox	Strophariaceae	*Agrocybe*			√	2	SS	HMJAU63516	
Amanita fulva	Amanitaceae	*Amanita*			√	1	EM	HMJAU64060	
Amanita sinicoflava	Amanitaceae	*Amanita*	√	√	√	6	EM	HMJAU64034	
Amanita submembranacea	Amanitaceae	*Amanita*	√		√	10	EM	HMJAU63766	OR364540
Ampulloclitocybe avellaneialba	Hygrophoraceae	*Ampulloclitocybe*			√	3	LS	HMJAU64008	
Ampulloclitocybe clavipes	Hygrophoraceae	*Ampulloclitocybe*			√	1	LS	HMJAU63996	OR364541
Armillaria cepistipes	Physalacriaceae	*Armillaria*		√	√	6	WS	HMJAU63593	OR364542
Armillaria gallica	Physalacriaceae	*Armillaria*	√	√	√	16	WS	HMJAU63565	OR364543
Armillaria mella	Physalacriaceae	*Armillaria*			√	1	WS	8202-8212	
Armillaria sinapina	Physalacriaceae	*Armillaria*		√	√	5	WS	HMJAU63568	
Armillaria tabescens	Physalacriaceae	*Armillaria*			√	1	WS	18449-18451	
Arrhenia epichysium	Arrhenieae	*Arrhenia*			√	1	WS	HMJAU63974	OR364544
Atheniella delectabilis	Arrhenieae	*Atheniella*			√	1	LS	HMJAU63931	OR364545
Auricularia heimuer	Auriculariaceae	*Auricularia*			√	1	WS	11639-11644	
Bjerkandera adusta	Phanerochaetaceae	*Bjerkandera*	√			2	WS	HMJAU63915	OR364546
Boletus edulis	Boletaceae	*Boletus*			√	1	EM	5993-6078	
Boletus kauffmanii	Boletaceae	*Boletus*			√	2	EM	HMJAU63601	
Bulgaria inguinans	Bulgariaceae	*Bulgaria*		√		1	WS	HMJAU63545	
Callistosporium luteo-olivaceum	Callistosporiaceae	*Callistosporium*	√		√	2	WS	HMJAU63935	OR364547
Calocera viscosa	Dacrymycetaceae	*Calocera*		√	√	2	WS	HMJAU63933	
Calocybe convexa	Lyophyllaceae	*Calocybe*			√	1	SS	HMJAU63491	
Campanella alba	Marasmiaceae	*Campanella*		√		1	WS	HMJAU63876	
Clavulina coralloides	Hydnaceae	*Clavulina*		√		1	EM	16249-16255	
Clavulina sphaeropedunculata	Hydnaceae	*Clavulina*		√		1	EM	HMJAU64003	

Clitocybe amarescens	Tricholomataceae	*Clitocybe*		√	√	3	LS	HMJAU63980	OR364548
Clitocybe dealbata	Tricholomataceae	*Clitocybe*	√	√		2	LS	HMJAU64005	
Clitocybe gibba	Tricholomataceae	*Clitocybe*			√	2	LS	5563-5591	
Clitocybe minutella	Tricholomataceae	*Clitocybe*			√	1	LS	HMJAU63530	
Clitocybe odora	Tricholomataceae	*Clitocybe*			√	1	LS	HMJAU63994	
Clitocybe phyllophila	Tricholomataceae	*Clitocybe*			√	1	LS	6079-6119	
Clitocybe sp1	Tricholomataceae	*Clitocybe*	√	√	√	6	LS	13587-13588	
Clitocybe sp2	Tricholomataceae	*Clitocybe*			√	1	LS	16705-16708	
Clitocybe sp3	Tricholomataceae	*Clitocybe*			√	1	LS	15771-15777	
Clitocybe vibecina	Tricholomataceae	*Clitocybe*	√	√	√	10	LS	HMJAU64044	OR364549
Conocybe moseri	Bolbitiaceae	*Conocybe*			√	2	LS	HMJAU63659	
Coprinellus xanthothrix	Psathyrellaceae	*Coprinellus*	√			2	SS	HMJAU63989	
Coprinopsis insignis	Psathyrellaceae	*Coprinopsis*	√		√	4	WS	HMJAU63844	OR364550
Coprinopsis jilinensis	Psathyrellaceae	*Coprinopsis*	√			2	WS	HMJAU63852	OR364551
Coprinopsis strossmayeri	Psathyrellaceae	*Coprinopsis*	√		√	2	WS	HMJAU63637	OR364552
Cortinarius cinnamomeus	Cortinariaceae	*Cortinarius*	√			2	EM	16855-16859	
Cortinarius hemitrichus	Cortinariaceae	*Cortinarius*	√			1	EM	20032-20040	
Cortinarius obtusus	Cortinariaceae	*Cortinarius*	√		√	5	EM	HMJAU63702	OR364553
Cortinarius olivaceoluteus	Cortinariaceae	*Cortinarius*	√	√	√	17	EM	HMJAU63751	OR364554
Cortinarius pellstonianus	Cortinariaceae	*Cortinarius*	√	√		3	EM	HMJAU64037	
Cortinarius semisanguineus	Cortinariaceae	*Cortinarius*	√	√	√	15	EM	HMJAU63584	
Cortinarius sommerfeltii	Cortinariaceae	*Cortinarius*		√		3	EM	HMJAU64027	
Cortinarius subcroceofolius	Cortinariaceae	*Cortinarius*		√		1	EM	20007-20013	
Cortinarius umbrinolens	Cortinariaceae	*Cortinarius*		√		1	EM	HMJAU63859	OR364555
Crepidotus mollis	Crepidotaceae	*Crepidotus*		√	√	4	WS	11960-11969	
Crepidotus sp1	Crepidotaceae	*Crepidotus*		√	√	2	WS	28168-28170	
Crepidotus sulphurinus	Crepidotaceae	*Crepidotus*		√	√	2	WS	13852-13857	
Crepidotus uber	Crepidotaceae	*Crepidotus*	√			1	WS	15837-15846	
Crinipellis scabella	Marasmiaceae	*Crinipellis*	√			1	WS	13177-13180	
Cystoderma amianthinum	Squamanitaceae	*Cystoderma*	√	√	√	13	SS	HMJAU63736	OR364556
Cystoderma muscicola	Squamanitaceae	*Cystoderma*	√	√	√	4	SS	HMJAU63991	OR364557
Dacryopinax spathularia	Dacrymycetaceae	*Dacryopinax*	√		√	2	WS	13223-13228	
Daldinia	Hypoxylaceae	*Daldinia*	√		√	6	WS	HMJAU63618	

concentrica									
Echinoderma asperum	Agaricaceae	*Echinoderma*	√		√	3	LS	HMJAU63518	OR364558
Elmerina holophaea	Aporpiaceae	*Elmerina*		√	√	3	WS	HMJAU63714	
Entocybe vinacea	Entolomataceae	*Entocybe*			√	1	WS	HMJAU64006	
Entoloma abortivum	Entolomataceae	*Entoloma*	√		√	5	SS	HMJAU63582	OR364559
Entoloma bisporigerum	Entolomataceae	*Entoloma*	√	√	√	8	SS	15783-15788	
Entoloma liaoningense	Entolomataceae	*Entoloma*	√			1	SS	17954-17963	
Galerina marginata	Strophariaceae	*Galerina*			√	1	WS	18462-18464	
Galerina sideroides	Strophariaceae	*Galerina*			√	1	WS	HMJAU64013	
Galerina stylifera	Strophariaceae	*Galerina*	√			1	WS	18562-18565	
Ganoderma applanatum	Ganodermataceae	*Ganoderma*	√		√	12	WS	HMJAU63631	OR364560
Geastrum fimbriatum	Geastraceae	*Geastrum*			√	2	LS	HMJAU63562	OR364561
Gymnopilus penetrans	Strophariaceae	*Gymnopilus*			√	1	WS	HMJAU63959	
Gymnopus confluens	Omphalotaceae	*Gymnopus*			√	1	LS	HMJAU63523	
Gymnopus dryophilus	Omphalotaceae	*Gymnopus*	√	√	√	45	LS	HMJAU63495	OR364562
Gymnopus polyphyllus	Omphalotaceae	*Gymnopus*	√	√	√	5	LS	11754-11767	OR364563
Gymnopus subnudus	Omphalotaceae	*Gymnopus*	√	√	√	22	LS	HMJAU63526	OR364564
Harmajaea guldeniae	Pseudoclitocybaceae	*Harmajaea*	√			1	LS	HMJAU64025	OR364565
Hebeloma marginatulum	Strophariaceae	*Hebeloma*	√			1	EM	HMJAU64041	
Hebeloma sordescens	Strophariaceae	*Hebeloma*	√	√		3	EM	HMJAU63866	OR364566
Hericium erinaceus	Hericiaceae	*Hericium*	√		√	15	WS	HMJAU63604	
Hohenbuehelia grisea	Pleurotaceae	*Hohenbuehelia*			√	1	WS	HMJAU63888	OR364567
Hypholoma capnoides	Strophariaceae	*Hypholoma*	√		√	3	WS	HMJAU64014	
Hypholoma fasciculare	Strophariaceae	*Hypholoma*	√	√	√	19	WS	HMJAU63550	OR364568
Hypsizygus ulmarius	Lyophyllaceae	*Hypsizygus*			√	1	WS	20191-20199	
Infundibulicybe gibba	Tricholomataceae	*Infundibulicybe*		√	√	8	SS	HMJAU63949	OR364569
Inocybe jucunda	Inocybaceae	*Inocybe*	√	√	√	8	SS	HMJAU64055	
Inocybe sororia	Inocybaceae	*Inocybe*	√	√	√	25	SS	HMJAU64023	OR364570
Inocybe sp1	Inocybaceae	*Inocybe*	√			1	SS	17289-17297	
Inocybe umbratica	Inocybaceae	*Inocybe*	√	√	√	4	SS	HMJAU63754	OR364571
Laccaria laccata	Hydnangiaceae	*Laccaria*		√	√	4	EM	HMJAU63997	OR364572
Lacrymaria	Psathyrellaceae	*Lacrymaria*		√		1	LS	HMJAU64018	

lacrymabunda									
Lactarius cremicolor	Russulaceae	*Lactarius*	√	√	√	25	EM	HMJAU63796	OR364573
Lactarius lanceolatus	Russulaceae	*Lactarius*	√			1	EM	19955-19959	
Lactarius picinus	Russulaceae	*Lactarius*	√	√	√	8	EM	HMJAU63833	OR364574
Lactarius sp1	Russulaceae	*Lactarius*		√	√	2	EM	17600-17607	
Lactarius tabidus	Russulaceae	*Lactarius*	√	√	√	18	EM	HMJAU63602	OR364575
Laetiporus sulphureus	Laetiporaceae	*Laetiporus*	√			1	WS	HMJAU63914	
Lentinellus cochleatus	Auriscalpiaceae	*Lentinellus*	√		√	8	WS	HMJAU63592	OR364576
Lentinula edodes	Omphalotaceae	*Lentinula*	√		√	3	WS	HMJAU63544	
Lepiota aspera	Agaricaceae	*Lepiota*			√	1	LS	17785-17789	
Lepiota cristata	Agaricaceae	*Lepiota*	√	√		3	LS	16080-16085	
Lepista nuda	Tricholomataceae	*Lepista*		√	√	10	SS	HMJAU63556	OR364577
Lycoperdon caudatum	Lycoperdaceae	*Lycoperdon*			√	2	SS	HMJAU63973	
Lycoperdon perlatum	Lycoperdaceae	*Lycoperdon*		√	√	2	SS	29523-29526	
Lyophyllum connatum	Lyophyllaceae	*Lyophyllum*			√	1	EM	HMJAU64009	OR364578
Lyophyllum decastes	Lyophyllaceae	*Lyophyllum*			√	1	EM	HMJAU63649	OR364579
Lyophyllum turcicum	Lyophyllaceae	*Lyophyllum*			√	2	EM	HMJAU63993	OR364580
Marasmiellus candidus	Omphalotaceae	*Marasmiellus*		√	√	3	LS	HMJAU63919	OR364581
Marasmiellus koreanus	Omphalotaceae	*Marasmiellus*			√	2	LS	HMJAU63656	OR364582
Marasmius cohaerens	Marasmiaceae	*Marasmius*	√		√	3	LS	HMJAU63939	OR364583
Marasmius insolitus	Marasmiaceae	*Marasmius*	√	√	√	5	LS	HMJAU63905	OR364584
Marasmius occultatiformis	Marasmiaceae	*Marasmius*	√	√	√	26	LS	HMJAU63583	OR364585
Marasmius ochroleucus	Marasmiaceae	*Marasmius*	√		√	5	LS	HMJAU63586	OR364586
Marasmius siccus	Marasmiaceae	*Marasmius*			√	2	LS	HMJAU63711	
Melanoleuca communis	Tricholomataceae	*Melanoleuca*			√	1	SS	HMJAU63934	OR364587
Melanoleuca leucopoda	Tricholomataceae	*Melanoleuca*	√	√	√	15	SS	HMJAU64011	OR364588
Mycena capillaripes	Mycenaceae	*Mycena*	√	√	√	6	LS	HMJAU63619	
Mycena galericulata	Mycenaceae	*Mycena*	√	√		2	LS	HMJAU64004	
Mycena laevigata	Mycenaceae	*Mycena*	√	√	√	3	LS	HMJAU63554	OR364589
Mycena lammiensis	Mycenaceae	*Mycena*	√			5	LS	HMJAU63689	OR364590
Mycena metata	Mycenaceae	*Mycena*	√	√	√	3	LS	16566-16575	
Mycena pearsoniana	Mycenaceae	*Mycena*	√	√	√	6	LS	HMJAU63764	OR364591

Mycena pelianthina	Mycenaceae	*Mycena*	√		√	6	LS	11342-11357	
Mycena polygramma	Mycenaceae	*Mycena*	√	√	√	12	LS	HMJAU63623	OR364592
Mycena pura	Mycenaceae	*Mycena*	√	√	√	39	LS	HMJAU63510	OR364593
Mycena purpureofusca	Mycenaceae	*Mycena*	√	√	√	6	LS	HMJAU63684	OR364594
Mycena quiniaultensis	Mycenaceae	*Mycena*		√	√	2	LS	HMJAU63972	OR364595
Mycena sp1	Mycenaceae	*Mycena*		√		1	LS	27794-27795	
Neolentinus lepideus	Gloeophyllaceae	*Neolentinus*	√	√	√	20	WS	HMJAU63515	OR364596
Notholepista fistulosa	Tricholomataceae	*Notholepista*			√	1	LS	HMJAU64050	
Onnia sp1	Hymenochaetaceae	*Onnia*			√	1	WS	13325-13329	
Onnia tomentosa	Hymenochaetaceae	*Onnia*	√	√	√	4	WS	HMJAU63917	OR364597
Ophiocordyceps nutans	Ophiocordycipitaceae	*Ophiocordyceps*			√	3	EI	HMJAU63522	OR364598
Otidea onotica	Pyronemataceae	*Otidea*		√	√	3	LS	HMJAU63860	OR364599
Oudemansiella mucida	Physalacriaceae	*Oudemansiella*	√	√	√	4	WS	HMJAU63757	OR364600
Panellus edulis	Mycenaceae	*Panellus*		√		1	WS	HMJAU63998	
Phallus flavocostatus	Phallaceae	*Phallus*	√			1	LS	28144-28145	
Pholiota lenta	Strophariaceae	*Pholiota*			√	1	WS	HMJAU63970	
Pholiota lubrica	Strophariaceae	*Pholiota*	√	√	√	17	WS	HMJAU64012	
Pholiota spumosa	Strophariaceae	*Pholiota*	√	√	√	10	WS	HMJAU63952	OR364601
Pholiota squarrosa	Strophariaceae	*Pholiota*	√			1	WS	HMJAU64039	
Picipes virgatus	Polyporaceae	*Picipes*	√	√		2	WS	HMJAU63925	
Pleurotus citrinopileatus	Pleurotaceae	*Pleurotus*	√	√	√	13	WS	HMJAU63683	
Pleurotus floridanus	Pleurotaceae	*Pleurotus*			√	1	WS	18516-18521	
Pleurotus ostreatus	Pleurotaceae	*Pleurotus*	√			3	WS	HMJAU63484	
Pleurotus pulmonarius	Pleurotaceae	*Pleurotus*	√			1	WS	HMJAU64061	
Pluteus cervinus	Pluteaceae	*Pluteus*	√			4	LS	HMJAU63694	
Pluteus hibbettii	Pluteaceae	*Pluteus*	√	√	√	11	LS	HMJAU63692	
Pluteus longistriatus	Pluteaceae	*Pluteus*	√	√		4	LS	HMJAU63487	
Pluteus podospileus	Pluteaceae	*Pluteus*			√	3	LS	HMJAU63646	OR364602
Pluteus pouzarianus	Pluteaceae	*Pluteus*		√		2	LS	HMJAU63661	
Pluteus velutinus	Pluteaceae	*Pluteus*			√	1	LS	HMJAU63645	OR364603
Porodaedalea chrysoloma	Hymenochaetaceae	*Porodaedalea*			√	1	WS	HMJAU64057	
Postia tephroleuca	Postiaceae	*Postia*			√	7	WS	HMJAU63643	
Psathyrella boreifasciculata	Psathyrellaceae	*Psathyrella*	√	√	√	9	WS	HMJAU63773	
Psathyrella	Psathyrellaceae	*Psathyrella*	√	√	√	19	WS	HMJAU63500	

candolleana									
Psathyrella sp1	Psathyrellaceae	*Psathyrella*		√		1	WS	10926-10945	
Pseudoomphalina intermedia	Tricholomataceae	*Pseudoomphalina*		√		1	LS	HMJAU63528	
Pseudosperma rimosum	Inocybaceae	*Pseudosperma*		√		1	SS	HMJAU64021	
Pterula multifida	Pterulaceae	*Pterula*		√		1	LS	16086-16094	
Ramaria apiculata	Gomphaceae	*Ramaria*		√		1	EM	28163-28164	
Ramaria comitis	Gomphaceae	*Ramaria*	√	√	√	60	EM	HMJAU63911	
Ramaria decurrens	Gomphaceae	*Ramaria*		√		3	EM	27807-27809	
Ramaria gracilioides	Gomphaceae	*Ramaria*			√	1	EM	HMJAU63520	
Rhodocollybia butyracea	Omphalotaceae	*Rhodocollybia*			√	2	SS	HMJAU63965	
Rigidoporus corticola	Meripilaceae	*Rigidoporus*			√	1	WS	HMJAU64040	
Russula aeruginea	Russulaceae	*Russula*	√		√	2	EM	HMJAU63837	
Russula delica	Russulaceae	*Russula*	√		√	2	EM	11413-11459	
Russula foetens	Russulaceae	*Russula*		√	√	11	EM	13755-13758	
Russula fragilis	Russulaceae	*Russula*		√		1	EM	5480-5512	
Russula madrensis	Russulaceae	*Russula*	√	√	√	3	EM	HMJAU63946	
Russula odorata	Russulaceae	*Russula*		√	√	4	EM	HMJAU63903	
Russula olivacea	Russulaceae	*Russula*		√	√	7	EM	8590-8609	
Russula puellaris	Russulaceae	*Russula*		√	√	5	EM	HMJAU63504	
Russula sanguinea	Russulaceae	*Russula*		√	√	5	EM	HMJAU63535	
Russula sororia	Russulaceae	*Russula*			√	1	EM	11544-11559	
Russula sp1	Russulaceae	*Russula*			√	1	EM	18526-18529	
Russula sp2	Russulaceae	*Russula*			√	4	EM	8756-8764	
Russula sp3	Russulaceae	*Russula*		√		1	EM	17594-17599	
Russula xerampelina	Russulaceae	*Russula*			√	1	EM	HMJAU63835	OR364604
Sarcoscypha coccinea	Sarcoscyphaceae	*Sarcoscypha*	√			1	WS	29939-29943	
Sarcoscypha dudleyi	Sarcoscyphaceae	*Sarcoscypha*			√	1	WS	HMJAU64017	
Schizophyllum commune	Schizophyllaceae	*Schizophyllum*			√	2	WS	HMJAU63634	
Singerocybe umbilicata	Tricholomataceae	*Singerocybe*			√	1	LS	HMJAU64001	
Sparassis latifolia	Sparassidaceae	*Sparassis*	√		√	7	EM	HMJAU63928	
Stereum hirsutum	Stereaceae	*Stereum*	√		√	3	WS	HMJAU63927	
Stereum subtomentosum	Stereaceae	*Stereum*	√		√	2	WS	HMJAU63891	
Stropharia aeruginosa	Strophariaceae	*Stropharia*	√			3	SS	HMJAU63977	
Tephrocybe ozes	Lyophyllaceae	*Tephrocybe*			√	1	LS	HMJAU64047	
Tephrocybe platypus	Lyophyllaceae	*Tephrocybe*		√		1	LS	HMJAU63850	
Thelephora palmata	Thelephoraceae	*Thelephora*		√		7	EM	HMJAU63727	OR364605
Trametes versicolor	Polyporaceae	*Trametes*	√	√	√	15	WS	HMJAU63548	
Trichaptum	Polyporaceae	*Trichaptum*	√	√	√	5	WS	HMJAU63883	

abietinum								
Trichaptum sp1	Polyporaceae	*Trichaptum*	√			1	WS	11951-11955
Trichaptum sp2	Polyporaceae	*Trichaptum*			√	1	WS	15832-15836
Tricholoma terreum	Tricholomataceae	*Tricholoma*	√	√		5	EM	HMJAU63983
Tubaria praestans	Tubariaceae	*Tubaria*	√			1	WS	HMJAU63893
Volvopluteus michiganensis	Pluteaceae	*Volvopluteus*	√			1	SS	HMJAU63693
Xeromphalina campanella	Mycenaceae	*Xeromphalina*		√	√	5	WS	28082-28085
Xeromphalina cauticinalis	Mycenaceae	*Xeromphalina*		√		2	WS	HMJAU63886
Xeromphalina enigmatica	Mycenaceae	*Xeromphalina*		√		3	WS	HMJAU63497
Xylaria hypoxylon	Xylariaceae	*Xylaria*	√		√	5	WS	HMJAU63638
Xylaria longipes	Xylariaceae	*Xylaria*	√			4	WS	HMJAU63651
Xylaria polymorpha	Xylariaceae	*Xylaria*	√			2	WS	HMJAU63878
Xylaria primorskensis	Xylariaceae	*Xylaria*		√		1	WS	HMJAU63896
Xylaria schweinitzii	Xylariaceae	*Xylaria*		√	√	2	WS	HMJAU63753

Note: Abbreviations: B = Broad-leaved forest; C = Coniferous forest; M = Mixed forest; N = Number of fruiting bodies; ML = Mode of Life; SN = Specimen Number; EM = ectomycorrhizal; SS = soil saprotroph; WS = wood saprotroph; LS = litter saprotroph; EI = endophyte-insect pathogen.

Table S2. Spearman correlation analysis of macrofungi and soil fungi.

Genus from macrofungi	Genus from soil	r	p-value	Relation
Xeromphalina	*Tricholoma_S*	0.974679434	0.00481823	positive
Xeromphalina	*Agrocybe_S*	0.974679434	0.00481823	positive
Crepidotus	*Entoloma_S*	-0.974679434	0.00481823	negative
Crepidotus	*Lentinellus_S*	0.917662935	0.028008456	positive
Xylaria	*Entoloma_S*	0.9	0.083333333	positive
Russula	*Tricholoma_S*	0.894427191	0.040519326	positive
Russula	*Pluteus_S*	0.894427191	0.040519326	positive
Russula	*Sparassis_S*	0.894427191	0.040519326	positive
Russula	*Agrocybe_S*	0.894427191	0.040519326	positive
Russula	*Lycoperdon_S*	0.894427191	0.040519326	positive
Pleurotus	*Sarcoscypha_S*	0.894427191	0.040519326	positive
Otidea	*Sarcoscypha_S*	0.894427191	0.040519326	positive
Pluteus	*Sarcoscypha_S*	0.894427191	0.040519326	positive
Trametes	*Tricholoma_S*	0.894427191	0.040519326	positive
Trametes	*Pluteus_S*	0.894427191	0.040519326	positive
Trametes	*Sparassis_S*	0.894427191	0.040519326	positive
Trametes	*Agrocybe_S*	0.894427191	0.040519326	positive
Trametes	*Lycoperdon_S*	0.894427191	0.040519326	positive
Peniophora	*Tricholoma_S*	0.894427191	0.040519326	positive
Peniophora	*Pluteus_S*	0.894427191	0.040519326	positive
Peniophora	*Sparassis_S*	0.894427191	0.040519326	positive
Peniophora	*Agrocybe_S*	0.894427191	0.040519326	positive
Peniophora	*Lycoperdon_S*	0.894427191	0.040519326	positive
Sparassis	*Crepidotus_S*	0.894427191	0.040519326	positive
Ophiocordyceps	*Sarcoscypha_S*	0.894427191	0.040519326	positive
Mucidula	*Sarcoscypha_S*	0.894427191	0.040519326	positive
Tephrocybe	*Tricholoma_S*	0.894427191	0.040519326	positive
Tephrocybe	*Pluteus_S*	0.894427191	0.040519326	positive
Tephrocybe	*Sparassis_S*	0.894427191	0.040519326	positive
Tephrocybe	*Agrocybe_S*	0.894427191	0.040519326	positive
Tephrocybe	*Lycoperdon_S*	0.894427191	0.040519326	positive
Lycoperdon	*Sarcoscypha_S*	0.894427191	0.040519326	positive
Russula	*Inocybe_S*	-0.894427191	0.040519326	negative
Xylaria	*Lentinellus_S*	-0.894427191	0.040519326	negative
Pleurotus	*Ramaria_S*	-0.894427191	0.040519326	negative
Pleurotus	*Otidea_S*	-0.894427191	0.040519326	negative
Pleurotus	*Crepidotus_S*	-0.894427191	0.040519326	negative
Otidea	*Ramaria_S*	-0.894427191	0.040519326	negative
Otidea	*Otidea_S*	-0.894427191	0.040519326	negative
Otidea	*Crepidotus_S*	-0.894427191	0.040519326	negative
Pluteus	*Ramaria_S*	-0.894427191	0.040519326	negative
Pluteus	*Otidea_S*	-0.894427191	0.040519326	negative
Pluteus	*Crepidotus_S*	-0.894427191	0.040519326	negative
Trametes	*Inocybe_S*	-0.894427191	0.040519326	negative
Peniophora	*Inocybe_S*	-0.894427191	0.040519326	negative
Ophiocordyceps	*Ramaria_S*	-0.894427191	0.040519326	negative
Ophiocordyceps	*Otidea_S*	-0.894427191	0.040519326	negative

Ophiocordyceps	*Crepidotus_S*	-0.894427191	0.040519326	negative
Mucidula	*Ramaria_S*	-0.894427191	0.040519326	negative
Mucidula	*Otidea_S*	-0.894427191	0.040519326	negative
Mucidula	*Crepidotus_S*	-0.894427191	0.040519326	negative
Tephrocybe	*Inocybe_S*	-0.894427191	0.040519326	negative
Lentinellus	*Entoloma_S*	-0.894427191	0.040519326	negative
Lycoperdon	*Ramaria_S*	-0.894427191	0.040519326	negative
Lycoperdon	*Otidea_S*	-0.894427191	0.040519326	negative
Lycoperdon	*Crepidotus_S*	-0.894427191	0.040519326	negative
Gymnopus	*Amanita_S*	-0.872081599	0.053854218	negative
Crepidotus	*Amanita_S*	-0.872081599	0.053854218	negative
Xeromphalina	*Mycena_S*	0.872081599	0.053854218	positive
Xeromphalina	*Ganoderma_S*	0.872081599	0.053854218	positive
Xeromphalina	*Pholiota_S*	0.872081599	0.053854218	positive
Gymnopus	*Ophiocordyceps_S*	-0.860309002	0.061343779	negative
Crepidotus	*Ophiocordyceps_S*	-0.860309002	0.061343779	negative
Gymnopus	*Sarcoscypha_S*	0.820782682	0.088587005	positive
Xeromphalina	*Pluteus_S*	0.820782682	0.088587005	positive
Xeromphalina	*Sparassis_S*	0.820782682	0.088587005	positive
Gymnopus	*Crepidotus_S*	-0.820782682	0.088587005	negative
Xeromphalina	*Inocybe_S*	-0.820782682	0.088587005	negative
Russula	*Stropharia_S*	0.802955069	0.10183797	positive
Trametes	*Stropharia_S*	0.802955069	0.10183797	positive
Peniophora	*Stropharia_S*	0.802955069	0.10183797	positive
Tephrocybe	*Stropharia_S*	0.802955069	0.10183797	positive
Xylaria	*Amanita_S*	0.8	0.133333333	positive
Mycena	*Entoloma_S*	-0.8	0.133333333	negative
Ramaria	*Entoloma_S*	-0.8	0.133333333	negative
Inocybe	*Xeromphalina_S*	0.790569415	0.111367155	positive
Inocybe	*Mucidula_S*	0.790569415	0.111367155	positive
Tricholoma	*Ophiocordyceps_S*	0.790569415	0.111367155	positive
Pleurotus	*Xeromphalina_S*	0.790569415	0.111367155	positive
Pleurotus	*Mucidula_S*	0.790569415	0.111367155	positive
Otidea	*Xeromphalina_S*	0.790569415	0.111367155	positive
Otidea	*Mucidula_S*	0.790569415	0.111367155	positive
Pluteus	*Xeromphalina_S*	0.790569415	0.111367155	positive
Pluteus	*Mucidula_S*	0.790569415	0.111367155	positive
Amanita	*Ophiocordyceps_S*	0.790569415	0.111367155	positive
Ganoderma	*Peniophora_S*	0.790569415	0.111367155	positive
Pholiota	*Xeromphalina_S*	0.790569415	0.111367155	positive
Pholiota	*Mucidula_S*	0.790569415	0.111367155	positive
Entoloma	*Ophiocordyceps_S*	0.790569415	0.111367155	positive
Ophiocordyceps	*Xeromphalina_S*	0.790569415	0.111367155	positive
Ophiocordyceps	*Mucidula_S*	0.790569415	0.111367155	positive
Mucidula	*Xeromphalina_S*	0.790569415	0.111367155	positive
Mucidula	*Mucidula_S*	0.790569415	0.111367155	positive
Stropharia	*Ophiocordyceps_S*	0.790569415	0.111367155	positive
Agrocybe	*Hypholoma_S*	0.790569415	0.111367155	positive
Lentinellus	*Pleurotus_S*	0.790569415	0.111367155	positive
Lentinellus	*Trametes_S*	0.790569415	0.111367155	positive
Lycoperdon	*Xeromphalina_S*	0.790569415	0.111367155	positive

Lycoperdon	*Mucidula_S*	0.790569415	0.111367155	positive
Sarcoscypha	*Ophiocordyceps_S*	0.790569415	0.111367155	positive
Russula	*Xylaria_S*	0.782623792	0.117613761	positive
Russula	*Mycena_S*	0.782623792	0.117613761	positive
Russula	*Psathyrella_S*	0.782623792	0.117613761	positive
Russula	*Ganoderma_S*	0.782623792	0.117613761	positive
Russula	*Pholiota_S*	0.782623792	0.117613761	positive
Xylaria	*Ophiocordyceps_S*	0.782623792	0.117613761	positive
Mycena	*Hypholoma_S*	0.782623792	0.117613761	positive
Ramaria	*Hypholoma_S*	0.782623792	0.117613761	positive
Pleurotus	*Tephrocybe_S*	0.782623792	0.117613761	positive
Otidea	*Tephrocybe_S*	0.782623792	0.117613761	positive
Pluteus	*Tephrocybe_S*	0.782623792	0.117613761	positive
Trametes	*Xylaria_S*	0.782623792	0.117613761	positive
Trametes	*Mycena_S*	0.782623792	0.117613761	positive
Trametes	*Psathyrella_S*	0.782623792	0.117613761	positive
Trametes	*Ganoderma_S*	0.782623792	0.117613761	positive
Trametes	*Pholiota_S*	0.782623792	0.117613761	positive
Peniophora	*Xylaria_S*	0.782623792	0.117613761	positive
Peniophora	*Mycena_S*	0.782623792	0.117613761	positive
Peniophora	*Psathyrella_S*	0.782623792	0.117613761	positive
Peniophora	*Ganoderma_S*	0.782623792	0.117613761	positive
Peniophora	*Pholiota_S*	0.782623792	0.117613761	positive
Sparassis	*Ramaria_S*	0.782623792	0.117613761	positive
Sparassis	*Otidea_S*	0.782623792	0.117613761	positive
Ophiocordyceps	*Tephrocybe_S*	0.782623792	0.117613761	positive
Mucidula	*Tephrocybe_S*	0.782623792	0.117613761	positive
Tephrocybe	*Xylaria_S*	0.782623792	0.117613761	positive
Tephrocybe	*Mycena_S*	0.782623792	0.117613761	positive
Tephrocybe	*Psathyrella_S*	0.782623792	0.117613761	positive
Tephrocybe	*Ganoderma_S*	0.782623792	0.117613761	positive
Tephrocybe	*Pholiota_S*	0.782623792	0.117613761	positive
Lentinellus	*Inocybe_S*	0.782623792	0.117613761	positive
Lycoperdon	*Tephrocybe_S*	0.782623792	0.117613761	positive
Inocybe	*Russula_S*	-0.782623792	0.117613761	negative
Mycena	*Ophiocordyceps_S*	-0.782623792	0.117613761	negative
Ramaria	*Ophiocordyceps_S*	-0.782623792	0.117613761	negative
Sparassis	*Tephrocybe_S*	-0.782623792	0.117613761	negative
Pholiota	*Russula_S*	-0.782623792	0.117613761	negative
Lentinellus	*Amanita_S*	-0.782623792	0.117613761	negative
Gymnopus	*Xeromphalina_S*	0.72547625	0.165370396	positive
Gymnopus	*Mucidula_S*	0.72547625	0.165370396	positive
Crepidotus	*Pleurotus_S*	0.72547625	0.165370396	positive
Crepidotus	*Trametes_S*	0.72547625	0.165370396	positive
Agrocybe	*Stropharia_S*	0.72547625	0.165370396	positive
Xeromphalina	*Xylaria_S*	0.718184846	0.171795305	positive
Crepidotus	*Inocybe_S*	0.718184846	0.171795305	positive
Xylaria	*Gymnopus_S*	0.707106781	0.181690114	positive
Xylaria	*Pleurotus_S*	-0.707106781	0.181690114	negative
Xylaria	*Trametes_S*	-0.707106781	0.181690114	negative
Mycena	*Gymnopus_S*	-0.707106781	0.181690114	negative

Mycena	*Pleurotus_S*	0.707106781	0.181690114	positive
Mycena	*Trametes_S*	0.707106781	0.181690114	positive
Ramaria	*Gymnopus_S*	-0.707106781	0.181690114	negative
Ramaria	*Pleurotus_S*	0.707106781	0.181690114	positive
Ramaria	*Trametes_S*	0.707106781	0.181690114	positive
Psathyrella	*Gymnopus_S*	-0.707106781	0.181690114	negative
Psathyrella	*Pleurotus_S*	0.707106781	0.181690114	positive
Psathyrella	*Trametes_S*	0.707106781	0.181690114	positive
Tricholoma	*Russula_S*	-0.707106781	0.181690114	negative
Tricholoma	*Inocybe_S*	-0.707106781	0.181690114	negative
Tricholoma	*Tricholoma_S*	0.707106781	0.181690114	positive
Tricholoma	*Pluteus_S*	0.707106781	0.181690114	positive
Tricholoma	*Amanita_S*	0.707106781	0.181690114	positive
Tricholoma	*Sparassis_S*	0.707106781	0.181690114	positive
Tricholoma	*Crepidotus_S*	0.707106781	0.181690114	positive
Tricholoma	*Entoloma_S*	0.707106781	0.181690114	positive
Tricholoma	*Agrocybe_S*	0.707106781	0.181690114	positive
Tricholoma	*Lycoperdon_S*	0.707106781	0.181690114	positive
Amanita	*Russula_S*	-0.707106781	0.181690114	negative
Amanita	*Inocybe_S*	-0.707106781	0.181690114	negative
Amanita	*Tricholoma_S*	0.707106781	0.181690114	positive
Amanita	*Pluteus_S*	0.707106781	0.181690114	positive
Amanita	*Amanita_S*	0.707106781	0.181690114	positive
Amanita	*Sparassis_S*	0.707106781	0.181690114	positive
Amanita	*Crepidotus_S*	0.707106781	0.181690114	positive
Amanita	*Entoloma_S*	0.707106781	0.181690114	positive
Amanita	*Agrocybe_S*	0.707106781	0.181690114	positive
Amanita	*Lycoperdon_S*	0.707106781	0.181690114	positive
Ganoderma	*Inocybe_S*	0.707106781	0.181690114	positive
Ganoderma	*Ramaria_S*	-0.707106781	0.181690114	negative
Ganoderma	*Otidea_S*	-0.707106781	0.181690114	negative
Ganoderma	*Amanita_S*	-0.707106781	0.181690114	negative
Ganoderma	*Crepidotus_S*	-0.707106781	0.181690114	negative
Ganoderma	*Sarcoscypha_S*	0.707106781	0.181690114	positive
Entoloma	*Russula_S*	-0.707106781	0.181690114	negative
Entoloma	*Inocybe_S*	-0.707106781	0.181690114	negative
Entoloma	*Tricholoma_S*	0.707106781	0.181690114	positive
Entoloma	*Pluteus_S*	0.707106781	0.181690114	positive
Entoloma	*Amanita_S*	0.707106781	0.181690114	positive
Entoloma	*Sparassis_S*	0.707106781	0.181690114	positive
Entoloma	*Crepidotus_S*	0.707106781	0.181690114	positive
Entoloma	*Entoloma_S*	0.707106781	0.181690114	positive
Entoloma	*Agrocybe_S*	0.707106781	0.181690114	positive
Entoloma	*Lycoperdon_S*	0.707106781	0.181690114	positive
Hypholoma	*Gymnopus_S*	-0.707106781	0.181690114	negative
Hypholoma	*Pleurotus_S*	0.707106781	0.181690114	positive
Hypholoma	*Trametes_S*	0.707106781	0.181690114	positive
Stropharia	*Russula_S*	-0.707106781	0.181690114	negative
Stropharia	*Inocybe_S*	-0.707106781	0.181690114	negative
Stropharia	*Tricholoma_S*	0.707106781	0.181690114	positive
Stropharia	*Pluteus_S*	0.707106781	0.181690114	positive

Stropharia	*Amanita_S*	0.707106781	0.181690114	positive
Stropharia	*Sparassis_S*	0.707106781	0.181690114	positive
Stropharia	*Crepidotus_S*	0.707106781	0.181690114	positive
Stropharia	*Entoloma_S*	0.707106781	0.181690114	positive
Stropharia	*Agrocybe_S*	0.707106781	0.181690114	positive
Stropharia	*Lycoperdon_S*	0.707106781	0.181690114	positive
Agrocybe	*Russula_S*	0.707106781	0.181690114	positive
Agrocybe	*Xylaria_S*	0.707106781	0.181690114	positive
Agrocybe	*Mycena_S*	0.707106781	0.181690114	positive
Agrocybe	*Psathyrella_S*	0.707106781	0.181690114	positive
Agrocybe	*Ganoderma_S*	0.707106781	0.181690114	positive
Agrocybe	*Pholiota_S*	0.707106781	0.181690114	positive
Agrocybe	*Tephrocybe_S*	0.707106781	0.181690114	positive
Sarcoscypha	*Russula_S*	-0.707106781	0.181690114	negative
Sarcoscypha	*Inocybe_S*	-0.707106781	0.181690114	negative
Sarcoscypha	*Tricholoma_S*	0.707106781	0.181690114	positive
Sarcoscypha	*Pluteus_S*	0.707106781	0.181690114	positive
Sarcoscypha	*Amanita_S*	0.707106781	0.181690114	positive
Sarcoscypha	*Sparassis_S*	0.707106781	0.181690114	positive
Sarcoscypha	*Crepidotus_S*	0.707106781	0.181690114	positive
Sarcoscypha	*Entoloma_S*	0.707106781	0.181690114	positive
Sarcoscypha	*Agrocybe_S*	0.707106781	0.181690114	positive
Sarcoscypha	*Lycoperdon_S*	0.707106781	0.181690114	positive
Psathyrella	*Ramaria_S*	0.7	0.233333333	positive
Psathyrella	*Tricholoma_S*	0.7	0.233333333	positive
Psathyrella	*Otidea_S*	0.7	0.233333333	positive
Psathyrella	*Agrocybe_S*	0.7	0.233333333	positive
Hypholoma	*Tephrocybe_S*	-0.7	0.233333333	negative
Pleurotus	*Amanita_S*	-0.670820393	0.215169943	negative
Otidea	*Amanita_S*	-0.670820393	0.215169943	negative
Pluteus	*Amanita_S*	-0.670820393	0.215169943	negative
Sparassis	*Russula_S*	-0.670820393	0.215169943	negative
Ophiocordyceps	*Amanita_S*	-0.670820393	0.215169943	negative
Mucidula	*Amanita_S*	-0.670820393	0.215169943	negative
Lentinellus	*Psathyrella_S*	-0.670820393	0.215169943	negative
Lentinellus	*Lycoperdon_S*	-0.670820393	0.215169943	negative
Lycoperdon	*Amanita_S*	-0.670820393	0.215169943	negative
Russula	*Amanita_S*	0.670820393	0.215169943	positive
Russula	*Entoloma_S*	0.670820393	0.215169943	positive
Inocybe	*Sarcoscypha_S*	0.670820393	0.215169943	positive
Mycena	*Lentinellus_S*	0.670820393	0.215169943	positive
Ramaria	*Lentinellus_S*	0.670820393	0.215169943	positive
Trametes	*Amanita_S*	0.670820393	0.215169943	positive
Trametes	*Entoloma_S*	0.670820393	0.215169943	positive
Peniophora	*Amanita_S*	0.670820393	0.215169943	positive
Peniophora	*Entoloma_S*	0.670820393	0.215169943	positive
Sparassis	*Tricholoma_S*	0.670820393	0.215169943	positive
Sparassis	*Agrocybe_S*	0.670820393	0.215169943	positive
Pholiota	*Sarcoscypha_S*	0.670820393	0.215169943	positive
Hypholoma	*Lentinellus_S*	0.670820393	0.215169943	positive
Tephrocybe	*Amanita_S*	0.670820393	0.215169943	positive

Tephrocybe	*Entoloma_S*	0.670820393	0.215169943	positive
Xeromphalina	*Lycoperdon_S*	0.666885929	0.218893981	positive
Gymnopus	*Ramaria_S*	-0.666885929	0.218893981	negative
Gymnopus	*Otidea_S*	-0.666885929	0.218893981	negative
Gymnopus	*Entoloma_S*	-0.666885929	0.218893981	negative
Xeromphalina	*Stropharia_S*	0.657894737	0.227470217	positive
Russula	*Lentinellus_S*	-0.625	0.259597198	negative
Inocybe	*Hypholoma_S*	-0.625	0.259597198	negative
Pleurotus	*Ophiocordyceps_S*	-0.625	0.259597198	negative
Otidea	*Ophiocordyceps_S*	-0.625	0.259597198	negative
Pluteus	*Ophiocordyceps_S*	-0.625	0.259597198	negative
Trametes	*Lentinellus_S*	-0.625	0.259597198	negative
Peniophora	*Lentinellus_S*	-0.625	0.259597198	negative
Pholiota	*Hypholoma_S*	-0.625	0.259597198	negative
Ophiocordyceps	*Ophiocordyceps_S*	-0.625	0.259597198	negative
Mucidula	*Ophiocordyceps_S*	-0.625	0.259597198	negative
Tephrocybe	*Lentinellus_S*	-0.625	0.259597198	negative
Lentinellus	*Ophiocordyceps_S*	-0.625	0.259597198	negative
Lycoperdon	*Ophiocordyceps_S*	-0.625	0.259597198	negative
Gymnopus	*Inocybe_S*	0.615587011	0.26899777	positive
Xeromphalina	*Crepidotus_S*	0.615587011	0.26899777	positive
Xylaria	*Inocybe_S*	-0.6	0.35	negative
Mycena	*Amanita_S*	-0.6	0.35	negative
Ramaria	*Amanita_S*	-0.6	0.35	negative
Psathyrella	*Mycena_S*	0.6	0.35	positive
Psathyrella	*Ganoderma_S*	0.6	0.35	positive
Psathyrella	*Pholiota_S*	0.6	0.35	positive
Psathyrella	*Crepidotus_S*	0.6	0.35	positive
Psathyrella	*Tephrocybe_S*	-0.6	0.35	negative
Hypholoma	*Russula_S*	-0.6	0.35	negative
Hypholoma	*Ramaria_S*	0.6	0.35	positive
Hypholoma	*Otidea_S*	0.6	0.35	positive
Gymnopus	*Tephrocybe_S*	0.564288094	0.321723336	positive
Xeromphalina	*Psathyrella_S*	0.564288094	0.321723336	positive
Xeromphalina	*Amanita_S*	0.564288094	0.321723336	positive
Crepidotus	*Lycoperdon_S*	-0.564288094	0.321723336	negative
Gymnopus	*Gymnopus_S*	-0.544107188	0.343110821	negative
Xeromphalina	*Gymnopus_S*	-0.544107188	0.343110821	negative
Xeromphalina	*Xeromphalina_S*	-0.544107188	0.343110821	negative
Xeromphalina	*Mucidula_S*	-0.544107188	0.343110821	negative
Crepidotus	*Gymnopus_S*	-0.544107188	0.343110821	negative
Lentinellus	*Stropharia_S*	-0.516185401	0.373252944	negative
Russula	*Ophiocordyceps_S*	0.5	0.391002219	positive
Pleurotus	*Peniophora_S*	0.5	0.391002219	positive
Otidea	*Peniophora_S*	0.5	0.391002219	positive
Pluteus	*Peniophora_S*	0.5	0.391002219	positive
Trametes	*Ophiocordyceps_S*	0.5	0.391002219	positive
Peniophora	*Ophiocordyceps_S*	0.5	0.391002219	positive
Sparassis	*Ophiocordyceps_S*	0.5	0.391002219	positive
Ophiocordyceps	*Peniophora_S*	0.5	0.391002219	positive
Mucidula	*Peniophora_S*	0.5	0.391002219	positive

Tephrocybe	*Ophiocordyceps_S*	0.5	0.391002219	positive
Lycoperdon	*Peniophora_S*	0.5	0.391002219	positive
Mycena	*Mycena_S*	0.5	0.45	positive
Mycena	*Ganoderma_S*	0.5	0.45	positive
Mycena	*Pholiota_S*	0.5	0.45	positive
Ramaria	*Mycena_S*	0.5	0.45	positive
Ramaria	*Ganoderma_S*	0.5	0.45	positive
Ramaria	*Pholiota_S*	0.5	0.45	positive
Hypholoma	*Tricholoma_S*	0.5	0.45	positive
Hypholoma	*Crepidotus_S*	0.5	0.45	positive
Hypholoma	*Agrocybe_S*	0.5	0.45	positive
Gymnopus	*Lentinellus_S*	0.458831468	0.436997621	positive
Pleurotus	*Stropharia_S*	0.458831468	0.436997621	positive
Otidea	*Stropharia_S*	0.458831468	0.436997621	positive
Pluteus	*Stropharia_S*	0.458831468	0.436997621	positive
Crepidotus	*Hypholoma_S*	0.458831468	0.436997621	positive
Ophiocordyceps	*Stropharia_S*	0.458831468	0.436997621	positive
Mucidula	*Stropharia_S*	0.458831468	0.436997621	positive
Lycoperdon	*Stropharia_S*	0.458831468	0.436997621	positive
Russula	*Crepidotus_S*	0.447213595	0.450184856	positive
Inocybe	*Ramaria_S*	-0.447213595	0.450184856	negative
Inocybe	*Otidea_S*	-0.447213595	0.450184856	negative
Inocybe	*Pluteus_S*	0.447213595	0.450184856	positive
Inocybe	*Sparassis_S*	0.447213595	0.450184856	positive
Inocybe	*Lycoperdon_S*	0.447213595	0.450184856	positive
Xylaria	*Hypholoma_S*	-0.447213595	0.450184856	negative
Psathyrella	*Hypholoma_S*	0.447213595	0.450184856	positive
Psathyrella	*Lentinellus_S*	0.447213595	0.450184856	positive
Pleurotus	*Inocybe_S*	0.447213595	0.450184856	positive
Pleurotus	*Xylaria_S*	0.447213595	0.450184856	positive
Pleurotus	*Psathyrella_S*	0.447213595	0.450184856	positive
Otidea	*Inocybe_S*	0.447213595	0.450184856	positive
Otidea	*Xylaria_S*	0.447213595	0.450184856	positive
Otidea	*Psathyrella_S*	0.447213595	0.450184856	positive
Pluteus	*Inocybe_S*	0.447213595	0.450184856	positive
Pluteus	*Xylaria_S*	0.447213595	0.450184856	positive
Pluteus	*Psathyrella_S*	0.447213595	0.450184856	positive
Trametes	*Crepidotus_S*	0.447213595	0.450184856	positive
Peniophora	*Crepidotus_S*	0.447213595	0.450184856	positive
Sparassis	*Inocybe_S*	-0.447213595	0.450184856	negative
Sparassis	*Pluteus_S*	0.447213595	0.450184856	positive
Sparassis	*Amanita_S*	0.447213595	0.450184856	positive
Sparassis	*Sparassis_S*	0.447213595	0.450184856	positive
Pholiota	*Ramaria_S*	-0.447213595	0.450184856	negative
Pholiota	*Otidea_S*	-0.447213595	0.450184856	negative
Pholiota	*Pluteus_S*	0.447213595	0.450184856	positive
Pholiota	*Sparassis_S*	0.447213595	0.450184856	positive
Pholiota	*Lycoperdon_S*	0.447213595	0.450184856	positive
Ophiocordyceps	*Inocybe_S*	0.447213595	0.450184856	positive
Ophiocordyceps	*Xylaria_S*	0.447213595	0.450184856	positive
Ophiocordyceps	*Psathyrella_S*	0.447213595	0.450184856	positive

Mucidula	*Inocybe_S*	0.447213595	0.450184856	positive
Mucidula	*Xylaria_S*	0.447213595	0.450184856	positive
Mucidula	*Psathyrella_S*	0.447213595	0.450184856	positive
Tephrocybe	*Crepidotus_S*	0.447213595	0.450184856	positive
Lentinellus	*Tephrocybe_S*	-0.447213595	0.450184856	negative
Lycoperdon	*Inocybe_S*	0.447213595	0.450184856	positive
Lycoperdon	*Xylaria_S*	0.447213595	0.450184856	positive
Lycoperdon	*Psathyrella_S*	0.447213595	0.450184856	positive
Xeromphalina	*Ramaria_S*	0.410391341	0.492535782	positive
Xeromphalina	*Otidea_S*	0.410391341	0.492535782	positive
Xeromphalina	*Entoloma_S*	0.410391341	0.492535782	positive
Crepidotus	*Psathyrella_S*	-0.410391341	0.492535782	negative
Xylaria	*Lycoperdon_S*	0.4	0.516666667	positive
Xylaria	*Sarcoscypha_S*	-0.4	0.516666667	negative
Psathyrella	*Pluteus_S*	0.4	0.516666667	positive
Psathyrella	*Sparassis_S*	0.4	0.516666667	positive
Hypholoma	*Entoloma_S*	-0.4	0.516666667	negative
Tricholoma	*Peniophora_S*	0.395284708	0.510140203	positive
Amanita	*Peniophora_S*	0.395284708	0.510140203	positive
Ganoderma	*Lentinellus_S*	0.395284708	0.510140203	positive
Sparassis	*Pleurotus_S*	0.395284708	0.510140203	positive
Sparassis	*Trametes_S*	0.395284708	0.510140203	positive
Entoloma	*Peniophora_S*	0.395284708	0.510140203	positive
Stropharia	*Peniophora_S*	0.395284708	0.510140203	positive
Lentinellus	*Xeromphalina_S*	0.395284708	0.510140203	positive
Lentinellus	*Mucidula_S*	0.395284708	0.510140203	positive
Sarcoscypha	*Peniophora_S*	0.395284708	0.510140203	positive
Russula	*Gymnopus_S*	-0.395284708	0.510140203	negative
Russula	*Pleurotus_S*	-0.395284708	0.510140203	negative
Russula	*Trametes_S*	-0.395284708	0.510140203	negative
Russula	*Xeromphalina_S*	-0.395284708	0.510140203	negative
Russula	*Mucidula_S*	-0.395284708	0.510140203	negative
Inocybe	*Gymnopus_S*	-0.395284708	0.510140203	negative
Inocybe	*Pleurotus_S*	-0.395284708	0.510140203	negative
Inocybe	*Trametes_S*	-0.395284708	0.510140203	negative
Tricholoma	*Hypholoma_S*	-0.395284708	0.510140203	negative
Tricholoma	*Lentinellus_S*	-0.395284708	0.510140203	negative
Pleurotus	*Gymnopus_S*	-0.395284708	0.510140203	negative
Pleurotus	*Pleurotus_S*	-0.395284708	0.510140203	negative
Pleurotus	*Trametes_S*	-0.395284708	0.510140203	negative
Otidea	*Gymnopus_S*	-0.395284708	0.510140203	negative
Otidea	*Pleurotus_S*	-0.395284708	0.510140203	negative
Otidea	*Trametes_S*	-0.395284708	0.510140203	negative
Pluteus	*Gymnopus_S*	-0.395284708	0.510140203	negative
Pluteus	*Pleurotus_S*	-0.395284708	0.510140203	negative
Pluteus	*Trametes_S*	-0.395284708	0.510140203	negative
Amanita	*Hypholoma_S*	-0.395284708	0.510140203	negative
Amanita	*Lentinellus_S*	-0.395284708	0.510140203	negative
Trametes	*Gymnopus_S*	-0.395284708	0.510140203	negative
Trametes	*Pleurotus_S*	-0.395284708	0.510140203	negative
Trametes	*Trametes_S*	-0.395284708	0.510140203	negative

Trametes	*Xeromphalina_S*	-0.395284708	0.510140203	negative
Trametes	*Mucidula_S*	-0.395284708	0.510140203	negative
Peniophora	*Gymnopus_S*	-0.395284708	0.510140203	negative
Peniophora	*Pleurotus_S*	-0.395284708	0.510140203	negative
Peniophora	*Trametes_S*	-0.395284708	0.510140203	negative
Peniophora	*Xeromphalina_S*	-0.395284708	0.510140203	negative
Peniophora	*Mucidula_S*	-0.395284708	0.510140203	negative
Ganoderma	*Hypholoma_S*	-0.395284708	0.510140203	negative
Ganoderma	*Ophiocordyceps_S*	-0.395284708	0.510140203	negative
Sparassis	*Gymnopus_S*	-0.395284708	0.510140203	negative
Sparassis	*Xeromphalina_S*	-0.395284708	0.510140203	negative
Sparassis	*Mucidula_S*	-0.395284708	0.510140203	negative
Pholiota	*Gymnopus_S*	-0.395284708	0.510140203	negative
Pholiota	*Pleurotus_S*	-0.395284708	0.510140203	negative
Pholiota	*Trametes_S*	-0.395284708	0.510140203	negative
Entoloma	*Hypholoma_S*	-0.395284708	0.510140203	negative
Entoloma	*Lentinellus_S*	-0.395284708	0.510140203	negative
Ophiocordyceps	*Gymnopus_S*	-0.395284708	0.510140203	negative
Ophiocordyceps	*Pleurotus_S*	-0.395284708	0.510140203	negative
Ophiocordyceps	*Trametes_S*	-0.395284708	0.510140203	negative
Mucidula	*Gymnopus_S*	-0.395284708	0.510140203	negative
Mucidula	*Pleurotus_S*	-0.395284708	0.510140203	negative
Mucidula	*Trametes_S*	-0.395284708	0.510140203	negative
Tephrocybe	*Gymnopus_S*	-0.395284708	0.510140203	negative
Tephrocybe	*Pleurotus_S*	-0.395284708	0.510140203	negative
Tephrocybe	*Trametes_S*	-0.395284708	0.510140203	negative
Tephrocybe	*Xeromphalina_S*	-0.395284708	0.510140203	negative
Tephrocybe	*Mucidula_S*	-0.395284708	0.510140203	negative
Stropharia	*Hypholoma_S*	-0.395284708	0.510140203	negative
Stropharia	*Lentinellus_S*	-0.395284708	0.510140203	negative
Agrocybe	*Peniophora_S*	-0.395284708	0.510140203	negative
Agrocybe	*Ophiocordyceps_S*	-0.395284708	0.510140203	negative
Agrocybe	*Lentinellus_S*	-0.395284708	0.510140203	negative
Lentinellus	*Gymnopus_S*	-0.395284708	0.510140203	negative
Lycoperdon	*Gymnopus_S*	-0.395284708	0.510140203	negative
Lycoperdon	*Pleurotus_S*	-0.395284708	0.510140203	negative
Lycoperdon	*Trametes_S*	-0.395284708	0.510140203	negative
Sarcoscypha	*Hypholoma_S*	-0.395284708	0.510140203	negative
Sarcoscypha	*Lentinellus_S*	-0.395284708	0.510140203	negative
Tricholoma	*Stropharia_S*	0.362738125	0.548485765	positive
Amanita	*Stropharia_S*	0.362738125	0.548485765	positive
Crepidotus	*Xeromphalina_S*	0.362738125	0.548485765	positive
Crepidotus	*Mucidula_S*	0.362738125	0.548485765	positive
Entoloma	*Stropharia_S*	0.362738125	0.548485765	positive
Stropharia	*Stropharia_S*	0.362738125	0.548485765	positive
Sarcoscypha	*Stropharia_S*	0.362738125	0.548485765	positive
Gymnopus	*Xylaria_S*	0.359092423	0.552814747	positive
Xylaria	*Xeromphalina_S*	-0.353553391	0.559404344	negative
Xylaria	*Mucidula_S*	-0.353553391	0.559404344	negative
Psathyrella	*Xeromphalina_S*	-0.353553391	0.559404344	negative
Psathyrella	*Mucidula_S*	-0.353553391	0.559404344	negative

Tricholoma	*Xylaria_S*	0.353553391	0.559404344	positive
Tricholoma	*Mycena_S*	0.353553391	0.559404344	positive
Tricholoma	*Ramaria_S*	0.353553391	0.559404344	positive
Tricholoma	*Psathyrella_S*	0.353553391	0.559404344	positive
Tricholoma	*Otidea_S*	0.353553391	0.559404344	positive
Tricholoma	*Ganoderma_S*	0.353553391	0.559404344	positive
Tricholoma	*Pholiota_S*	0.353553391	0.559404344	positive
Tricholoma	*Tephrocybe_S*	-0.353553391	0.559404344	negative
Amanita	*Xylaria_S*	0.353553391	0.559404344	positive
Amanita	*Mycena_S*	0.353553391	0.559404344	positive
Amanita	*Ramaria_S*	0.353553391	0.559404344	positive
Amanita	*Psathyrella_S*	0.353553391	0.559404344	positive
Amanita	*Otidea_S*	0.353553391	0.559404344	positive
Amanita	*Ganoderma_S*	0.353553391	0.559404344	positive
Amanita	*Pholiota_S*	0.353553391	0.559404344	positive
Amanita	*Tephrocybe_S*	-0.353553391	0.559404344	negative
Ganoderma	*Russula_S*	-0.353553391	0.559404344	negative
Ganoderma	*Mycena_S*	-0.353553391	0.559404344	negative
Ganoderma	*Tricholoma_S*	-0.353553391	0.559404344	negative
Ganoderma	*Ganoderma_S*	-0.353553391	0.559404344	negative
Ganoderma	*Pholiota_S*	-0.353553391	0.559404344	negative
Ganoderma	*Entoloma_S*	-0.353553391	0.559404344	negative
Ganoderma	*Tephrocybe_S*	0.353553391	0.559404344	positive
Ganoderma	*Agrocybe_S*	-0.353553391	0.559404344	negative
Entoloma	*Xylaria_S*	0.353553391	0.559404344	positive
Entoloma	*Mycena_S*	0.353553391	0.559404344	positive
Entoloma	*Ramaria_S*	0.353553391	0.559404344	positive
Entoloma	*Psathyrella_S*	0.353553391	0.559404344	positive
Entoloma	*Otidea_S*	0.353553391	0.559404344	positive
Entoloma	*Ganoderma_S*	0.353553391	0.559404344	positive
Entoloma	*Pholiota_S*	0.353553391	0.559404344	positive
Entoloma	*Tephrocybe_S*	-0.353553391	0.559404344	negative
Stropharia	*Xylaria_S*	0.353553391	0.559404344	positive
Stropharia	*Mycena_S*	0.353553391	0.559404344	positive
Stropharia	*Ramaria_S*	0.353553391	0.559404344	positive
Stropharia	*Psathyrella_S*	0.353553391	0.559404344	positive
Stropharia	*Otidea_S*	0.353553391	0.559404344	positive
Stropharia	*Ganoderma_S*	0.353553391	0.559404344	positive
Stropharia	*Pholiota_S*	0.353553391	0.559404344	positive
Stropharia	*Tephrocybe_S*	-0.353553391	0.559404344	negative
Agrocybe	*Inocybe_S*	-0.353553391	0.559404344	negative
Agrocybe	*Ramaria_S*	-0.353553391	0.559404344	negative
Agrocybe	*Tricholoma_S*	0.353553391	0.559404344	positive
Agrocybe	*Otidea_S*	-0.353553391	0.559404344	negative
Agrocybe	*Pluteus_S*	0.353553391	0.559404344	positive
Agrocybe	*Sparassis_S*	0.353553391	0.559404344	positive
Agrocybe	*Crepidotus_S*	-0.353553391	0.559404344	negative
Agrocybe	*Agrocybe_S*	0.353553391	0.559404344	positive
Agrocybe	*Lycoperdon_S*	0.353553391	0.559404344	positive
Agrocybe	*Sarcoscypha_S*	0.353553391	0.559404344	positive
Sarcoscypha	*Xylaria_S*	0.353553391	0.559404344	positive

Sarcoscypha	*Mycena_S*	0.353553391	0.559404344	positive
Sarcoscypha	*Ramaria_S*	0.353553391	0.559404344	positive
Sarcoscypha	*Psathyrella_S*	0.353553391	0.559404344	positive
Sarcoscypha	*Otidea_S*	0.353553391	0.559404344	positive
Sarcoscypha	*Ganoderma_S*	0.353553391	0.559404344	positive
Sarcoscypha	*Pholiota_S*	0.353553391	0.559404344	positive
Sarcoscypha	*Tephrocybe_S*	-0.353553391	0.559404344	negative
Gymnopus	*Peniophora_S*	0.344123601	0.570656465	positive
Gymnopus	*Hypholoma_S*	0.344123601	0.570656465	positive
Xeromphalina	*Hypholoma_S*	0.344123601	0.570656465	positive
Xeromphalina	*Ophiocordyceps_S*	0.344123601	0.570656465	positive
Xeromphalina	*Lentinellus_S*	-0.344123601	0.570656465	negative
Pleurotus	*Entoloma_S*	-0.335410197	0.581090673	negative
Otidea	*Entoloma_S*	-0.335410197	0.581090673	negative
Pluteus	*Entoloma_S*	-0.335410197	0.581090673	negative
Sparassis	*Mycena_S*	0.335410197	0.581090673	positive
Sparassis	*Ganoderma_S*	0.335410197	0.581090673	positive
Sparassis	*Pholiota_S*	0.335410197	0.581090673	positive
Ophiocordyceps	*Entoloma_S*	-0.335410197	0.581090673	negative
Mucidula	*Entoloma_S*	-0.335410197	0.581090673	negative
Lentinellus	*Xylaria_S*	-0.335410197	0.581090673	negative
Lentinellus	*Pluteus_S*	-0.335410197	0.581090673	negative
Lentinellus	*Sparassis_S*	-0.335410197	0.581090673	negative
Lycoperdon	*Entoloma_S*	-0.335410197	0.581090673	negative
Crepidotus	*Crepidotus_S*	-0.307793506	0.614384003	negative
Crepidotus	*Sarcoscypha_S*	0.307793506	0.614384003	positive
Xylaria	*Psathyrella_S*	0.3	0.683333333	positive
Mycena	*Inocybe_S*	0.3	0.683333333	positive
Mycena	*Xylaria_S*	0.3	0.683333333	positive
Mycena	*Tricholoma_S*	0.3	0.683333333	positive
Mycena	*Agrocybe_S*	0.3	0.683333333	positive
Mycena	*Lycoperdon_S*	-0.3	0.683333333	negative
Mycena	*Sarcoscypha_S*	0.3	0.683333333	positive
Ramaria	*Inocybe_S*	0.3	0.683333333	positive
Ramaria	*Xylaria_S*	0.3	0.683333333	positive
Ramaria	*Tricholoma_S*	0.3	0.683333333	positive
Ramaria	*Agrocybe_S*	0.3	0.683333333	positive
Ramaria	*Lycoperdon_S*	-0.3	0.683333333	negative
Ramaria	*Sarcoscypha_S*	0.3	0.683333333	positive
Psathyrella	*Russula_S*	-0.3	0.683333333	negative
Psathyrella	*Xylaria_S*	0.3	0.683333333	positive
Psathyrella	*Entoloma_S*	-0.3	0.683333333	negative
Hypholoma	*Mycena_S*	0.3	0.683333333	positive
Hypholoma	*Psathyrella_S*	-0.3	0.683333333	negative
Hypholoma	*Pluteus_S*	0.3	0.683333333	positive
Hypholoma	*Ganoderma_S*	0.3	0.683333333	positive
Hypholoma	*Sparassis_S*	0.3	0.683333333	positive
Hypholoma	*Pholiota_S*	0.3	0.683333333	positive
Gymnopus	*Stropharia_S*	0.289473684	0.636644755	positive
Tricholoma	*Gymnopus_S*	-0.25	0.685037642	negative
Tricholoma	*Pleurotus_S*	-0.25	0.685037642	negative

Tricholoma	*Trametes_S*	-0.25	0.685037642	negative
Tricholoma	*Xeromphalina_S*	-0.25	0.685037642	negative
Tricholoma	*Mucidula_S*	-0.25	0.685037642	negative
Amanita	*Gymnopus_S*	-0.25	0.685037642	negative
Amanita	*Pleurotus_S*	-0.25	0.685037642	negative
Amanita	*Trametes_S*	-0.25	0.685037642	negative
Amanita	*Xeromphalina_S*	-0.25	0.685037642	negative
Amanita	*Mucidula_S*	-0.25	0.685037642	negative
Ganoderma	*Gymnopus_S*	-0.25	0.685037642	negative
Ganoderma	*Pleurotus_S*	-0.25	0.685037642	negative
Ganoderma	*Trametes_S*	-0.25	0.685037642	negative
Entoloma	*Gymnopus_S*	-0.25	0.685037642	negative
Entoloma	*Pleurotus_S*	-0.25	0.685037642	negative
Entoloma	*Trametes_S*	-0.25	0.685037642	negative
Entoloma	*Xeromphalina_S*	-0.25	0.685037642	negative
Entoloma	*Mucidula_S*	-0.25	0.685037642	negative
Stropharia	*Gymnopus_S*	-0.25	0.685037642	negative
Stropharia	*Pleurotus_S*	-0.25	0.685037642	negative
Stropharia	*Trametes_S*	-0.25	0.685037642	negative
Stropharia	*Xeromphalina_S*	-0.25	0.685037642	negative
Stropharia	*Mucidula_S*	-0.25	0.685037642	negative
Agrocybe	*Gymnopus_S*	-0.25	0.685037642	negative
Agrocybe	*Pleurotus_S*	-0.25	0.685037642	negative
Agrocybe	*Trametes_S*	-0.25	0.685037642	negative
Agrocybe	*Xeromphalina_S*	-0.25	0.685037642	negative
Agrocybe	*Mucidula_S*	-0.25	0.685037642	negative
Sarcoscypha	*Gymnopus_S*	-0.25	0.685037642	negative
Sarcoscypha	*Pleurotus_S*	-0.25	0.685037642	negative
Sarcoscypha	*Trametes_S*	-0.25	0.685037642	negative
Sarcoscypha	*Xeromphalina_S*	-0.25	0.685037642	negative
Sarcoscypha	*Mucidula_S*	-0.25	0.685037642	negative
Crepidotus	*Stropharia_S*	-0.236842105	0.701286739	negative
Inocybe	*Stropharia_S*	0.229415734	0.710481725	positive
Pholiota	*Stropharia_S*	0.229415734	0.710481725	positive
Russula	*Sarcoscypha_S*	0.223606798	0.717685644	positive
Inocybe	*Inocybe_S*	0.223606798	0.717685644	positive
Inocybe	*Xylaria_S*	0.223606798	0.717685644	positive
Inocybe	*Psathyrella_S*	0.223606798	0.717685644	positive
Pleurotus	*Pluteus_S*	0.223606798	0.717685644	positive
Pleurotus	*Sparassis_S*	0.223606798	0.717685644	positive
Pleurotus	*Lycoperdon_S*	0.223606798	0.717685644	positive
Otidea	*Pluteus_S*	0.223606798	0.717685644	positive
Otidea	*Sparassis_S*	0.223606798	0.717685644	positive
Otidea	*Lycoperdon_S*	0.223606798	0.717685644	positive
Pluteus	*Pluteus_S*	0.223606798	0.717685644	positive
Pluteus	*Sparassis_S*	0.223606798	0.717685644	positive
Pluteus	*Lycoperdon_S*	0.223606798	0.717685644	positive
Trametes	*Sarcoscypha_S*	0.223606798	0.717685644	positive
Peniophora	*Sarcoscypha_S*	0.223606798	0.717685644	positive
Sparassis	*Entoloma_S*	0.223606798	0.717685644	positive
Sparassis	*Lycoperdon_S*	0.223606798	0.717685644	positive

Pholiota	*Inocybe_S*	0.223606798	0.717685644	positive
Pholiota	*Xylaria_S*	0.223606798	0.717685644	positive
Pholiota	*Psathyrella_S*	0.223606798	0.717685644	positive
Hypholoma	*Peniophora_S*	0.223606798	0.717685644	positive
Ophiocordyceps	*Pluteus_S*	0.223606798	0.717685644	positive
Ophiocordyceps	*Sparassis_S*	0.223606798	0.717685644	positive
Ophiocordyceps	*Lycoperdon_S*	0.223606798	0.717685644	positive
Mucidula	*Pluteus_S*	0.223606798	0.717685644	positive
Mucidula	*Sparassis_S*	0.223606798	0.717685644	positive
Mucidula	*Lycoperdon_S*	0.223606798	0.717685644	positive
Tephrocybe	*Sarcoscypha_S*	0.223606798	0.717685644	positive
Lentinellus	*Ramaria_S*	0.223606798	0.717685644	positive
Lentinellus	*Otidea_S*	0.223606798	0.717685644	positive
Lycoperdon	*Pluteus_S*	0.223606798	0.717685644	positive
Lycoperdon	*Sparassis_S*	0.223606798	0.717685644	positive
Lycoperdon	*Lycoperdon_S*	0.223606798	0.717685644	positive
Russula	*Russula_S*	-0.223606798	0.717685644	negative
Inocybe	*Amanita_S*	-0.223606798	0.717685644	negative
Inocybe	*Crepidotus_S*	-0.223606798	0.717685644	negative
Mycena	*Peniophora_S*	-0.223606798	0.717685644	negative
Ramaria	*Peniophora_S*	-0.223606798	0.717685644	negative
Trametes	*Russula_S*	-0.223606798	0.717685644	negative
Peniophora	*Russula_S*	-0.223606798	0.717685644	negative
Sparassis	*Sarcoscypha_S*	-0.223606798	0.717685644	negative
Pholiota	*Amanita_S*	-0.223606798	0.717685644	negative
Pholiota	*Crepidotus_S*	-0.223606798	0.717685644	negative
Tephrocybe	*Russula_S*	-0.223606798	0.717685644	negative
Lentinellus	*Russula_S*	-0.223606798	0.717685644	negative
Lentinellus	*Mycena_S*	-0.223606798	0.717685644	negative
Lentinellus	*Tricholoma_S*	-0.223606798	0.717685644	negative
Lentinellus	*Ganoderma_S*	-0.223606798	0.717685644	negative
Lentinellus	*Pholiota_S*	-0.223606798	0.717685644	negative
Lentinellus	*Agrocybe_S*	-0.223606798	0.717685644	negative
Gymnopus	*Psathyrella_S*	0.20519567	0.740581942	positive
Xeromphalina	*Russula_S*	-0.20519567	0.740581942	negative
Crepidotus	*Pluteus_S*	-0.20519567	0.740581942	negative
Crepidotus	*Sparassis_S*	-0.20519567	0.740581942	negative
Xylaria	*Mycena_S*	-0.2	0.783333333	negative
Xylaria	*Ganoderma_S*	-0.2	0.783333333	negative
Xylaria	*Pholiota_S*	-0.2	0.783333333	negative
Xylaria	*Crepidotus_S*	0.2	0.783333333	positive
Xylaria	*Tephrocybe_S*	0.2	0.783333333	positive
Mycena	*Russula_S*	0.2	0.783333333	positive
Mycena	*Ramaria_S*	0.2	0.783333333	positive
Mycena	*Otidea_S*	0.2	0.783333333	positive
Ramaria	*Russula_S*	0.2	0.783333333	positive
Ramaria	*Ramaria_S*	0.2	0.783333333	positive
Ramaria	*Otidea_S*	0.2	0.783333333	positive
Psathyrella	*Inocybe_S*	-0.2	0.783333333	negative
Hypholoma	*Amanita_S*	-0.2	0.783333333	negative
Gymnopus	*Russula_S*	0.153896753	0.804828817	positive

Gymnopus	*Mycena_S*	0.153896753	0.804828817	positive
Gymnopus	*Ganoderma_S*	0.153896753	0.804828817	positive
Gymnopus	*Pholiota_S*	0.153896753	0.804828817	positive
Xeromphalina	*Tephrocybe_S*	-0.153896753	0.804828817	negative
Crepidotus	*Tephrocybe_S*	-0.153896753	0.804828817	negative
Russula	*Peniophora_S*	0.125	0.8412605	positive
Russula	*Hypholoma_S*	0.125	0.8412605	positive
Inocybe	*Ophiocordyceps_S*	0.125	0.8412605	positive
Inocybe	*Lentinellus_S*	0.125	0.8412605	positive
Pleurotus	*Hypholoma_S*	0.125	0.8412605	positive
Pleurotus	*Lentinellus_S*	0.125	0.8412605	positive
Otidea	*Hypholoma_S*	0.125	0.8412605	positive
Otidea	*Lentinellus_S*	0.125	0.8412605	positive
Pluteus	*Hypholoma_S*	0.125	0.8412605	positive
Pluteus	*Lentinellus_S*	0.125	0.8412605	positive
Trametes	*Peniophora_S*	0.125	0.8412605	positive
Trametes	*Hypholoma_S*	0.125	0.8412605	positive
Peniophora	*Peniophora_S*	0.125	0.8412605	positive
Peniophora	*Hypholoma_S*	0.125	0.8412605	positive
Sparassis	*Peniophora_S*	0.125	0.8412605	positive
Sparassis	*Hypholoma_S*	-0.125	0.8412605	negative
Sparassis	*Lentinellus_S*	0.125	0.8412605	positive
Pholiota	*Ophiocordyceps_S*	0.125	0.8412605	positive
Pholiota	*Lentinellus_S*	0.125	0.8412605	positive
Ophiocordyceps	*Hypholoma_S*	0.125	0.8412605	positive
Ophiocordyceps	*Lentinellus_S*	0.125	0.8412605	positive
Mucidula	*Hypholoma_S*	0.125	0.8412605	positive
Mucidula	*Lentinellus_S*	0.125	0.8412605	positive
Tephrocybe	*Peniophora_S*	0.125	0.8412605	positive
Tephrocybe	*Hypholoma_S*	0.125	0.8412605	positive
Lentinellus	*Peniophora_S*	0.125	0.8412605	positive
Lentinellus	*Hypholoma_S*	0.125	0.8412605	positive
Lycoperdon	*Hypholoma_S*	0.125	0.8412605	positive
Lycoperdon	*Lentinellus_S*	0.125	0.8412605	positive
Inocybe	*Mycena_S*	-0.111803399	0.857944618	negative
Inocybe	*Ganoderma_S*	-0.111803399	0.857944618	negative
Inocybe	*Pholiota_S*	-0.111803399	0.857944618	negative
Xylaria	*Peniophora_S*	-0.111803399	0.857944618	negative
Psathyrella	*Peniophora_S*	-0.111803399	0.857944618	negative
Psathyrella	*Ophiocordyceps_S*	-0.111803399	0.857944618	negative
Pleurotus	*Tricholoma_S*	-0.111803399	0.857944618	negative
Pleurotus	*Agrocybe_S*	-0.111803399	0.857944618	negative
Otidea	*Tricholoma_S*	-0.111803399	0.857944618	negative
Otidea	*Agrocybe_S*	-0.111803399	0.857944618	negative
Pluteus	*Tricholoma_S*	-0.111803399	0.857944618	negative
Pluteus	*Agrocybe_S*	-0.111803399	0.857944618	negative
Sparassis	*Psathyrella_S*	-0.111803399	0.857944618	negative
Pholiota	*Mycena_S*	-0.111803399	0.857944618	negative
Pholiota	*Ganoderma_S*	-0.111803399	0.857944618	negative
Pholiota	*Pholiota_S*	-0.111803399	0.857944618	negative
Hypholoma	*Ophiocordyceps_S*	-0.111803399	0.857944618	negative

Ophiocordyceps	*Tricholoma_S*	-0.111803399	0.857944618	negative
Ophiocordyceps	*Agrocybe_S*	-0.111803399	0.857944618	negative
Mucidula	*Tricholoma_S*	-0.111803399	0.857944618	negative
Mucidula	*Agrocybe_S*	-0.111803399	0.857944618	negative
Lentinellus	*Crepidotus_S*	-0.111803399	0.857944618	negative
Lycoperdon	*Tricholoma_S*	-0.111803399	0.857944618	negative
Lycoperdon	*Agrocybe_S*	-0.111803399	0.857944618	negative
Russula	*Ramaria_S*	0.111803399	0.857944618	positive
Russula	*Otidea_S*	0.111803399	0.857944618	positive
Russula	*Tephrocybe_S*	0.111803399	0.857944618	positive
Inocybe	*Tricholoma_S*	0.111803399	0.857944618	positive
Inocybe	*Entoloma_S*	0.111803399	0.857944618	positive
Inocybe	*Tephrocybe_S*	0.111803399	0.857944618	positive
Inocybe	*Agrocybe_S*	0.111803399	0.857944618	positive
Pleurotus	*Russula_S*	0.111803399	0.857944618	positive
Pleurotus	*Mycena_S*	0.111803399	0.857944618	positive
Pleurotus	*Ganoderma_S*	0.111803399	0.857944618	positive
Pleurotus	*Pholiota_S*	0.111803399	0.857944618	positive
Otidea	*Russula_S*	0.111803399	0.857944618	positive
Otidea	*Mycena_S*	0.111803399	0.857944618	positive
Otidea	*Ganoderma_S*	0.111803399	0.857944618	positive
Otidea	*Pholiota_S*	0.111803399	0.857944618	positive
Pluteus	*Russula_S*	0.111803399	0.857944618	positive
Pluteus	*Mycena_S*	0.111803399	0.857944618	positive
Pluteus	*Ganoderma_S*	0.111803399	0.857944618	positive
Pluteus	*Pholiota_S*	0.111803399	0.857944618	positive
Trametes	*Ramaria_S*	0.111803399	0.857944618	positive
Trametes	*Otidea_S*	0.111803399	0.857944618	positive
Trametes	*Tephrocybe_S*	0.111803399	0.857944618	positive
Peniophora	*Ramaria_S*	0.111803399	0.857944618	positive
Peniophora	*Otidea_S*	0.111803399	0.857944618	positive
Peniophora	*Tephrocybe_S*	0.111803399	0.857944618	positive
Sparassis	*Xylaria_S*	0.111803399	0.857944618	positive
Pholiota	*Tricholoma_S*	0.111803399	0.857944618	positive
Pholiota	*Entoloma_S*	0.111803399	0.857944618	positive
Pholiota	*Tephrocybe_S*	0.111803399	0.857944618	positive
Pholiota	*Agrocybe_S*	0.111803399	0.857944618	positive
Hypholoma	*Hypholoma_S*	0.111803399	0.857944618	positive
Ophiocordyceps	*Russula_S*	0.111803399	0.857944618	positive
Ophiocordyceps	*Mycena_S*	0.111803399	0.857944618	positive
Ophiocordyceps	*Ganoderma_S*	0.111803399	0.857944618	positive
Ophiocordyceps	*Pholiota_S*	0.111803399	0.857944618	positive
Mucidula	*Russula_S*	0.111803399	0.857944618	positive
Mucidula	*Mycena_S*	0.111803399	0.857944618	positive
Mucidula	*Ganoderma_S*	0.111803399	0.857944618	positive
Mucidula	*Pholiota_S*	0.111803399	0.857944618	positive
Tephrocybe	*Ramaria_S*	0.111803399	0.857944618	positive
Tephrocybe	*Otidea_S*	0.111803399	0.857944618	positive
Tephrocybe	*Tephrocybe_S*	0.111803399	0.857944618	positive
Lentinellus	*Sarcoscypha_S*	0.111803399	0.857944618	positive
Lycoperdon	*Russula_S*	0.111803399	0.857944618	positive

Lycoperdon	*Mycena_S*	0.111803399	0.857944618	positive
Lycoperdon	*Ganoderma_S*	0.111803399	0.857944618	positive
Lycoperdon	*Pholiota_S*	0.111803399	0.857944618	positive
Gymnopus	*Tricholoma_S*	-0.102597835	0.869597921	negative
Gymnopus	*Agrocybe_S*	-0.102597835	0.869597921	negative
Crepidotus	*Tricholoma_S*	-0.102597835	0.869597921	negative
Crepidotus	*Agrocybe_S*	-0.102597835	0.869597921	negative
Hypholoma	*Stropharia_S*	-0.102597835	0.869597921	negative
Xylaria	*Stropharia_S*	0.102597835	0.869597921	positive
Mycena	*Stropharia_S*	0.102597835	0.869597921	positive
Ramaria	*Stropharia_S*	0.102597835	0.869597921	positive
Psathyrella	*Stropharia_S*	0.102597835	0.869597921	positive
Gymnopus	*Pluteus_S*	0.102597835	0.869597921	positive
Gymnopus	*Sparassis_S*	0.102597835	0.869597921	positive
Xeromphalina	*Sarcoscypha_S*	0.102597835	0.869597921	positive
Xylaria	*Russula_S*	0.1	0.95	positive
Xylaria	*Xylaria_S*	-0.1	0.95	negative
Xylaria	*Ramaria_S*	-0.1	0.95	negative
Xylaria	*Tricholoma_S*	-0.1	0.95	negative
Xylaria	*Otidea_S*	-0.1	0.95	negative
Xylaria	*Agrocybe_S*	-0.1	0.95	negative
Mycena	*Psathyrella_S*	-0.1	0.95	negative
Mycena	*Pluteus_S*	0.1	0.95	positive
Mycena	*Sparassis_S*	0.1	0.95	positive
Mycena	*Crepidotus_S*	-0.1	0.95	negative
Mycena	*Tephrocybe_S*	-0.1	0.95	negative
Ramaria	*Psathyrella_S*	-0.1	0.95	negative
Ramaria	*Pluteus_S*	0.1	0.95	positive
Ramaria	*Sparassis_S*	0.1	0.95	positive
Ramaria	*Crepidotus_S*	-0.1	0.95	negative
Ramaria	*Tephrocybe_S*	-0.1	0.95	negative
Psathyrella	*Psathyrella_S*	-0.1	0.95	negative
Hypholoma	*Inocybe_S*	0.1	0.95	positive
Hypholoma	*Xylaria_S*	0.1	0.95	positive
Hypholoma	*Lycoperdon_S*	-0.1	0.95	negative
Hypholoma	*Sarcoscypha_S*	0.1	0.95	positive
Xeromphalina	*Peniophora_S*	-0.057353933	0.927014759	negative
Gymnopus	*Lycoperdon_S*	-0.051298918	0.934712848	negative
Crepidotus	*Russula_S*	0.051298918	0.934712848	positive
Crepidotus	*Xylaria_S*	-0.051298918	0.934712848	negative
Crepidotus	*Mycena_S*	0.051298918	0.934712848	positive
Crepidotus	*Ramaria_S*	0.051298918	0.934712848	positive
Crepidotus	*Otidea_S*	0.051298918	0.934712848	positive
Crepidotus	*Ganoderma_S*	0.051298918	0.934712848	positive
Crepidotus	*Pholiota_S*	0.051298918	0.934712848	positive
